# Significance of plasma MACC1 levels on the prognostic stratification in patients with colorectal cancer

**DOI:** 10.1111/jcmm.13989

**Published:** 2018-10-28

**Authors:** Aifen Lin, Rui‐Li Zhang, Xia Zhang, Xiao‐Fang He, Jian‐Gang Zhang, Wei‐Hua Yan

**Affiliations:** ^1^ Biological Resource Center Taizhou Hospital of Zhejiang Province Wenzhou Medical University Linhai Zhejiang China; ^2^ Department of Gastrointestinal Surgery Taizhou Hospital of Zhejiang Province Wenzhou Medical University Linhai Zhejiang China; ^3^ Department of Laboratory Medicine Lanxi Peoples's Hospital Lanxi Zhejiang China; ^4^ Medical Research Center Taizhou Hospital of Zhejiang Province Wenzhou Medical University Linhai Zhejiang China

**Keywords:** colorectal cancer, diagnosis, MACC1, prognosis

## Abstract

The clinical significance of metastasis‐associated in colon cancer‐1 (MACC1) has been investigated but the relevance of peripheral MACC1 levels was rather limited. Herein, our data revealed that plasma MACC1 levels in 117 colorectal cancer patients (CRC) were dramatically higher than that in normal controls (*P* < 0.001), and with a strong discrimination power between the two groups (AUC = 0.960, *P* < 0.001). Moreover, MACC1 is an independent prognostic factor for CRC patients. When clinical parameters stratified by MACC1_low_ and MACC1_high_, MACC1 levels exhibited further significant predictive value. Summary, plasma MACC1 levels could be a useful prognostic and diagnostic biomarker, and could improve the prognostic value of traditional prognosticators for colorectal cancer patients.

## 1 INTRODUCTION

Metastasis‐ associated in colon cancer 1 (MACC1), identified in colon cancer patients in 2009 for the first time, has been found to play multiple important roles in tumourigenesis and metastasis.^1^

In colorectal cancer patients (CRC), lesion MACC1 expression has been observed to be notably higher in tumours, and higher levels of MACC1 expression were remarkably associated with tumour metastasis and patient worse prognosis.^2^, ^3^ In addition to its clinical significance in CRC patients, the prognostic and diagnostic value of MACC1 was further solidified later in other malignancies such as hepatocellular cancer,^4^ gastric cancer.^5^ However, clinical relevance of peripheral plasma MACC1 levels was rather limited.

In this study, plasma MACC1 in 117 CRC patients were analysed with ELISA, and its clinical significance was evaluated.

## 2 MATERIALS AND METHODS

### 2.1 CRC patients

In total,117 consecutive CRC pre‐operative plasma were included from April 2007 to May 2013 at Taizhou Hospital of Zhejiang province (National human genetic resources platform of China YCZYPT [2017]02). Clinical stage was according to the AJCC 7th TNM staging system.^6^ Patient's overall survival was defined from the data of surgical operation to the last follow‐up.

Written informed consent was obtained from each participant prior to the surgery, and this study was approved by the Institutional Ethics Review Board of Taizhou Hospital of Zhejiang Province.

### 2.2 MACC1 enzyme‐linked immunosorbent assay (ELISA)

Plasma MACC1 detection was performed with the MACC1 ELISA kit (Aviva Systems Biology, Corp., San Diego, CA, USA). Samples were measured in duplicates. Details of the performance were according to the manufacture's protocol. Briefly, 100 μL of serially titrated standards and CRC plasma were added to a 96‐well microplate coated with MACC1 antibody. Then, biotinylated‐MACC1 detector antibody and avidin‐HRP conjugate were added and incubated. 3,3′,5,5′‐tetramethylbenzidine substrate was added and the reaction was terminated with stop solution. Finally, the optical density was read with a microplate reader at 450 nm (Spectra Max 250; Molecular Devices, Sunnyvale, CA, USA). The concentration of MACC1 was determined by optical density according to the standard curves.

### 2.3 Statistical analysis

Group MACC1 comparison was analysed with Mann‐Whitney *U*‐test. Receiver operating characteristics (ROC) analysis was performed and the cut‐off value was determined by Youden's index. Survival probabilities were evaluated with Kaplan‐Meier method, and differences in survival were analysed by the log‐rank test. Statistical analysis was performed with SPSS v.13.0 (SPSS, Inc., Chicago, IL, USA). All statistical tests were two‐sided and *P *< 0.05 was considered statistically significant.

## 3 RESULTS

### 3.1 Relationship between plasma MACC1 levels and clinical variables in CRC patients

**Figure 1:**
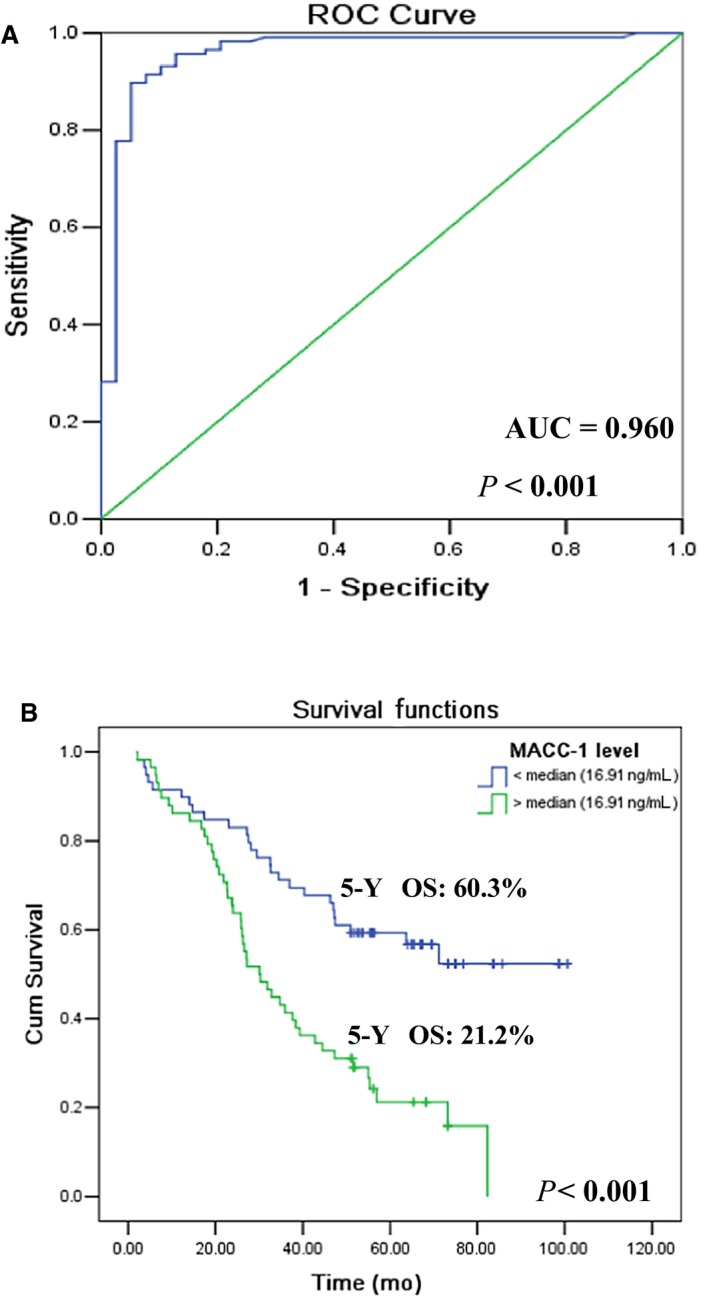
A, ROC curve analysis for the performance of plasma MACC1 to distinguish CRC patients from normal controls. B, Comparison of the overall survival between the CRC patients with plasma MACC1_high_ (n = 58) and MACC1_low_ (n = 58) by Kaplan‐Meier survival analysis

Plasma MACC1 levels in CRC patients and normal controls and comparison between the groups were detailed in Table S1. The median levels of MACC1 in CRC patients were notably increased than normal controls (16.91 ng/mL vs 1.51 ng/mL; *P *< 0.001), which could effectively distinguish the CRC patients from normal controls with the ROC curve (AUC = 0.960; *P *< 0.001). An optimal cut‐off value was determined with the Youden's index for MACC1 was 3.43 ng/ml, with the sensitivity (0.897) and specificity (0.948; Figure 1A).

In CRC patients, no significant association was found for MACC1 levels to gender, age, and primary tumour status (T). However, MACC1 levels were significantly associated with regional lymph node status (N). MACC1 in patients with N_0_, N_1_ and N_2_ was 15.56 ng/mL, 20.02 ng/mL and 23.43 ng/mL respectively (*P *= 0.010). Much higher MACC1 levels were also observed in patients with M_1_ than those with M_0_ (45.21 ng/mL vs 16.77 ng/mL; *P *= 0.034), and in patients with AJCC_III+IV_ than those with AJCC_I+II_ (22.13 ng/mL vs 14.30 ng/mL; *P *= 0.004). Moreover, MACC1 levels in died CRC patients were dramatically higher than that in live CRC patients (25.99 ng/mL vs 10.84 ng/mL; *P *< 0.001; Table** **S1).

### 3.2 Plasma MACC1 levels are associated with CRC patient survival

To analyse the prognostic impact of plasma MACC1 levels for CRC patients, MACC1 levels were divided into two groups according to the median level (16.91 ng/mL) as MACC1_low_ (<16.91 ng/mL) or MACC1_high_ (>16.91 ng/mL). Data showed that CRC patients with MACC1_high_ had a much worse survival than those with MACC1_low_ (median: 38.7 months vs 68.1 months*, P *< 0.001), and much lower 5‐year survival rate (21.2% vs 60.3%; *P *< 0.001; Figure 1B). Moreover, a worse survival was observed between patients with age above median (>67 years) vs younger (*P *= 0.037), N_1+2_ vs N_0_ (*P *< 0.001) and AJCC_III+IV_ vs AJCC_I+II_ (*P *< 0.001). However, no statistical difference was observed between male vs female (*P *= 0.509), T_3+4_ vs T_2_ (*P *= 0.508). Although the survival in patients with M_1_ (n = 3) was much less than that with M_0_ (n = 113), no significance was observed (*P *= 0.158), which may be caused by only three M_1_ patients were included in the study (Table 1).

**Table 1:** Log‐rank Mantel‐Cox analysis of stratified variables in survival by plasma MACC1 levels in CRC patients

Variables	Stratified variables	Whole cohort				MACC1<16.9 ng/ml			MACC1 >16.9 ng/ml			*P*
		No. total	No. events	Survival time Mean (95% CI)	*P*	No. total	No. events	Survival time Mean (95% CI)	No. total	No. events	Survival time Mean (95% CI)	
Gender	Male	69	40	55.1 (46.3‐63.9)	0.509	36	15	66.6 (54.3‐79.0)	33	25	41.5 (32.2‐50.8)	0.011
	Female	47	31	52.2 (41.9‐62.4)		22	10	69.3 (54.7‐83.9)	25	21	34.5 (25.4‐43.6)	0.002
Age	≤67 y	56	29	61.5 (51.6‐71.4)	0.037	33	12	74.4 (62.4‐86.4)	23	17	41.4 (29.4‐53.5)	0.002
	>67 y	60	42	47.6 (38.9‐56.4)		25	13	59.6 (44.7‐74.4)	35	29	35.4 (28.6‐42.1)	0.034
Tumour status	T_2_	14	8	60.4 (42.6‐78.2)	0.508	9	4	74.6 (57.7‐91.6)	5	4	30.5 (11.8‐49.2)	0.013
	T_3+4_	102	63	54.6 (47.2‐62.0)		49	21	70.7 (60.0‐81.4)	53	42	39.2 (32.2‐46.2)	<0.001
Nodal status	N_0_	44	16	72.2 (61.6‐82.8)	<0.001	27	7	80.8 (69.1‐92.5)	17	9	47.0 (34.8‐59.1)	0.033
	N_1+2_	72	55	44.3 (36.7‐51.9)		31	18	58.1 (44.8‐71.4)	41	37	34.5 (27.5‐41.6)	0.006
Metastasis status	M_0_	113	68	55.4 (48.5‐62.4)	0.158	58	25	69.2 (59.6‐78.8)	55	43	39.1 (32.1‐46.2)	<0.001
	M_1_	3	3	31.2 (22.9‐39.5)		/	/	/	3	3	31.2 (22.9‐39.5)	/
Disease stage	I+II	42	14	73.9 (63.1‐84.7)	<0.001	27	7	80.8 (69.1‐92.5)	15	7	48.5 (34.9‐62.1)	0.093
	III+IV	74	57	44.0 (48.0‐61.6)		31	18	58.1 (44.8‐71.4)	43	39	34.6 (27.9‐41.3)	0.005

Among these variables, Cox's proportional hazards model analysis showed that plasma MACC1 levels is an independent prognostic marker for CRC patients (HR = 2.121, *P *= 0.004; Table S2).

### 3.3 Plasma MACC1 levels impact on the prognostic stratification of clinical variables in CRC patients

To be noted, previous studies have reported unconventional risk factors such as log odds of positive lymph nodes (LODDS) or MACC1 levels could improve the prognostic power of the stratified risk factors in CRC patients.^7^, ^8^ Herein, we evaluate the impact of plasma MACC1 levels on the prognostic stratification of clinical variables (Table 1).

Data revealed that MACC1 levels are of great significance in survival when CRC clinical parameters were stratified. Parameters such as patient gender and T stage alone were not significantly related to the survival. However, when stratified into subgroups, such as male (*P *= 0.011) or female (*P *= 0.002) patients with MACC1_high_ have a significantly worse survival than patients with MACC1_low_. Similarly, patients with MACC1_high_ have a significant prognostic power for patients with T_2_ (*P *= 0.013) and T_3+4_ (*P *< 0.001). Other factors such as age, M status, N status and AJCC stage were all of evident statistical significance when those variables were stratified.

## 4 DISCUSSION

A wealth of studies have been carried out and strengthen the potential application of both MACC1 transcripts and protein expression as a novel diagnostic and prognostic indicator in various malignancies.^9^, ^10^ However, “liquid biopsies” such as peripheral circulating cell‐free nucleic acids and soluble proteins, being minimal invasive, economical and routinely applicable, their special interest has emerged. In this context, Stein et al firstly reported that circulating MACC1 transcripts is associated with metastasis and prognosis in CRC patients, where high MACC1 transcript levels are correlated with worse survival.^11^

In this study, our data showed plasma MACC1 levels were markedly elevated in CRC patients, which could powerfully discriminate the CRC patients from normal controls. With the cut‐off value of 3.43 ng/mL determined by Youden's index, the sensitivity and specificity was 0.897 and 0.948 respectively. In breast cancer, Tan et al 12 reported that, with the cut‐off value 59.05 pg/mL (AUC = 0.757), the sensitivity and specificity were 0.714 and 0.891 respectively. Though has a similar diagnostic power, the cut‐off value between our study and that in Tan et al^12^ varies dramatically. This may be caused by the different samples used for MACC1 detection, the methodology with different ELISA Kit, and more importantly, the different cancer types analysed. Therefore, more studies are necessary to explore and evaluate the clinical significance of the circulating MACC1 protein among different types of cancers.

Moreover, higher plasma MACC1 levels were positively correlated to the tumour status of N_1_ or N_2_, M_1_ and AJCC_III+IV_, and also in died CRC patients. These findings were consistent with previous reports in patients with ovarian cancer and pancreatic cancer.^13^, ^14^ Our results further revealed that patients with MACC1_high_ had an obviously worse survival and a 5‐year survival rate compared with those with MACC1_low_, and plasma MACC1 was an significant independent prognostic biomarker for CRC patients, strengthening the issue that MACC1 was a novel and reliable molecule in prognostic prediction as earlier studies emphasized.^1^, ^10^

Finally, we evaluate whether MACC1 levels in stratified clinical parameters could improve their prognostic power in CRC patients. Data showed that patients with MACC1_high_ has much worse survival than those with MACC1_low_ in the male or female, younger or elder patients. This trend was also observed in subgroup patients with M_0_, N_0_, N_1_, T_2_, T_3+4_ and stage AJCC_III+IV_.

In conclusion, our study revealed that plasma MACC1 is a clinical relevant biomarker in diagnosis and prognosis in CRC, and the incorporation of MACC1 levels with other stratified clinical parameters could improve their prognostic values for CRC subpopulations.

## CONFLICT OF INTERESTS

The authors declared no conflicts of interest.
