# Supplementary material for: Significance of plasma MACC1 levels on the prognostic stratification in patients with colorectal cancer
Source: J Cell Mol Med. 2018 Oct 28;23(2):1598–601. doi: 10.1111/jcmm.13989 (PMC6349203; doi:10.1111/jcmm.13989)
Supplement: Supplementary file 1 [file JCMM-23-1598-s001.doc]

| **Suppl. Table 1** Association of plasma MACC1 levels with clinicopathological parameters in colorectal cancer patients | | | |
| --- | --- | --- | --- |
| Variables | No. | MACC1 median (range, ng/ml) | *p***^*^** |
| Normal controls | 40 | 1.51 (1.23 – 30.98) | <0.001 |
| CRC patients | 117 | 16.91 (1.26 – 187.57) |
| Gender |  |  |  |
| male | 69 | 16.77 (1.26 – 166.49) | 0.786 |
| female | 48 | 19.26 (1.68 – 187.57) |
| Age |  |  |  |
| ≤median (67 yrs) | 57 | 16.33 (1.26– 187.57) | 0.084 |
| >median | 60 | 18.89 (2.03 – 166.49) |
| T category |  |  |  |
| T_2_ | 14 | 12.02 (2.03– 62.69) | 0.393 |
| T_3+4_ | 103 | 17.61 (1.26– 187.57) |
| N category |  |  |  |
| N_0_ | 44 | 15.56 (1.26– 164.02) | 0.010 |
| N_1_ | 42 | 20.02 (1.68– 187.57) |
| N_2_ | 31 | 23.43 (3.06– 126.27) |
| M category |  |  |  |
| M_0_ | 113 | 16.77 (1.26– 187.57) | 0.034 |
| M_1_ | 4 | 45.21 (26.54– 64.07) |
| Disease stage |  |  |  |
| I+ II | 42 | 14.30 (1.26– 164.02) | 0.004 |
| III+ IV | 75 | 22.13 (1.68– 187.57) |
| Survival status |  |  |  |
| dead | 45 | 10.84 (1.26– 45.08) | <0.001 |
| alive | 71 | 25.99 (1.68– 187.57) |
| **^*^** Comparison of sHLA-G expression status between or among each variable using the Mann-Whitney U test. | | | |
