# Supplementary material for: Significance of plasma MACC1 levels on the prognostic stratification in patients with colorectal cancer
Source: J Cell Mol Med. 2018 Oct 28;23(2):1598–601. doi: 10.1111/jcmm.13989 (PMC6349203; doi:10.1111/jcmm.13989)
Supplement: Supplementary file 2 [file JCMM-23-1598-s002.doc]

| **Suppl.Table 2** Cox proportional hazards model analysis of variables affecting overall survival in colorectal cancer patients | | | | | | | |
| --- | --- | --- | --- | --- | --- | --- | --- |
| Variables | Categories |  | Univariate Analysis | |  | Multivariate Analysis | |
|  |  |  | HR (95% CI) | *P*-value |  | HR (95% CI) | *P*-value |
| Gender | Male (*vs* female) |  | 1.171 (0.732 – 1.873) | 0.510 |  | / |  |
| Age (years) | >67 (*vs* ≤67) |  | 1.655 (1.027 – 2.669) | 0.039 |  | 1.674 (1.019 – 2.750) | 0.042 |
| T category | T_3+4_ (*vs* T_1+2_) |  | 1.282 (0.614 – 2.678) | 0.509 |  | / |  |
| N category | N_1+2_ (*vs* N_0_) |  | 2.894 (1.654 – 5.061) | <0.001 |  | 1.319 (0.312 – 5.568) | 0.706 |
| M category | M_1_ (*vs* M_0_) |  | 2.267 (0.705 – 7.288) | 0.169 |  | / |  |
| Disease stage | III/IV (*vs* I/II) |  | 3.231 (1.796 – 5.815) | <0.001 |  | 2.259 (0.495 – 10.31) | 0.293 |
| MACC1 (ng/ml) | >16.9 (*vs* ≤16.9) |  | 2.785 (1.698 – 4.568) | <0.001 |  | 2.121 (1.274 – 3.531) | 0.004 |
| Abbreviations: HR=hazard ratio; 95% CI=95% confidence interval; TNM, lymph-node-metastasis and disease stage. | | | | | | | |
